# Dihydro­allocryptopine

**DOI:** 10.1107/S1600536811052172

**Published:** 2011-12-10

**Authors:** Wenwen Sun, Yuyan Qin, Zhe Hou, Yao Yao, Le Zhou

**Affiliations:** a College of Science, Northwest Agriculture and Forestry University, Yangling 712100, People’s Republic of China

## Abstract

In the title compound [systematic name: 7,8-dimeth­oxy-11-methyl-17,19-dioxa-11-aza­tetra­cyclo­[12.7.0.0^4,9^.0^16,20^]henicosa-1(21),4,6,8,14,16 (20)-hexaen-2-ol], C_21_H_25_NO_5_, the benzene rings are inclined at a dihedral angle of 23.16 (5)°. One of the meth­oxy C atoms is close to coplanar with its attached ring [deviation = 0.129 (3) Å], whereas the other is orientated away from the ring [deviation = −1.124 (2) Å]. The 10-membered ring is highly puckered, and the OH and CH_3_ substituents project to the same side of the ring. In the crystal, O—H⋯O hydrogen bonds link the mol­ecules into [010] chains and C—H⋯O and C—H⋯π inter­actions consolidate the packing.

## Related literature

For the synthesis of the title compound, see: Wada *et al.* (2007 ▶). For the biological activity of allocryptopine derivatives, see: Morteza *et al.* (2003 ▶); Yan *et al.* (2009 ▶); Capasso *et al.* (1997 ▶); Jeong *et al.* (2009 ▶); Zhao *et al.* (2008 ▶). For a related structure, see: Valpuesta *et al.* (2006 ▶).
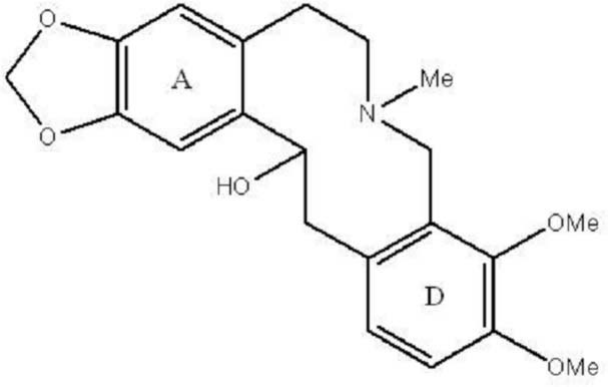


## Experimental

### 

#### Crystal data

C_21_H_25_NO_5_
*M* *_r_* = 371.42
Monoclinic, 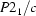

*a* = 14.2557 (19) Å
*b* = 9.3705 (13) Å
*c* = 15.278 (2) Å
β = 106.601 (2)°
*V* = 1955.8 (5) Å^3^
*Z* = 4
Mo *K*α radiation
μ = 0.09 mm^−1^
*T* = 296 K
0.45 × 0.24 × 0.21 mm

#### Data collection

Bruker SMART APEX II CCD diffractometer
Absorption correction: multi-scan (*SADABS*; Sheldrick, 1996 ▶) *T*_min_ = 0.961, *T*_max_ = 0.981
14189 measured reflections
3646 independent reflections
2766 reflections with *I* > 2σ(*I*)
*R*_int_ = 0.026

#### Refinement

*R*[*F* ^2^ > 2σ(*F* ^2^)] = 0.041
*wR*(*F* ^2^) = 0.110
*S* = 1.02
3646 reflections
248 parameters
H-atom parameters constrained
Δρ_max_ = 0.18 e Å^−3^
Δρ_min_ = −0.21 e Å^−3^

### 

Data collection: *APEX2* (Bruker, 2004 ▶); cell refinement: *SAINT* (Bruker, 2004 ▶); data reduction: *SAINT*; program(s) used to solve structure: *SHELXS97* (Sheldrick, 2008 ▶); program(s) used to refine structure: *SHELXL97* (Sheldrick, 2008 ▶); molecular graphics: *SHELXTL* (Sheldrick, 2008 ▶); software used to prepare material for publication: *SHELXTL*.

## Supplementary Material

Crystal structure: contains datablock(s) global, I. DOI: 10.1107/S1600536811052172/hb6507sup1.cif

Structure factors: contains datablock(s) I. DOI: 10.1107/S1600536811052172/hb6507Isup2.hkl

Supplementary material file. DOI: 10.1107/S1600536811052172/hb6507Isup3.cml

Additional supplementary materials:  crystallographic information; 3D view; checkCIF report

## Figures and Tables

**Table 1:** Hydrogen-bond geometry (Å, °) *Cg*2 is the centroid of the C1–C6 benzene ring.

*D*—H⋯*A*	*D*—H	H⋯*A*	*D*⋯*A*	*D*—H⋯*A*
O3—H3⋯O5^i^	0.82	2.03	2.8380 (16)	168
C7—H7*A*⋯O4^ii^	0.97	2.57	3.405 (3)	144
C18—H18⋯O3^iii^	0.93	2.53	3.229 (2)	132
C7—H7*B*⋯*Cg*2^iv^	0.97	2.57	3.464 (3)	153

Symmetry codes: (i) 
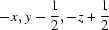
; (ii) 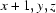; (iii) 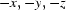; (iv) 
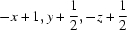
.

**Fig. 1.**
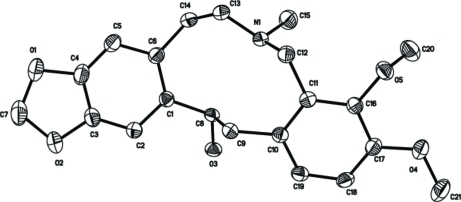
The molecular structure of the title compound (50% displacement ellipsoids).

**Fig. 2.**
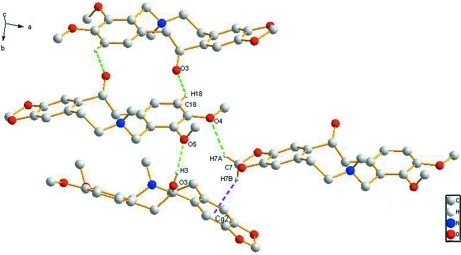
The two-dimension sheet structure of the title compound.

## supplementary crystallographic information

### Comment

The allocryptopine derivatives have recently attracted great attention due to their antifungal activity (Morteza *et al.* 2003), antibacterial activity (Yan *et al.* 2009), analgesic effect (Capasso *et al.* 1997), anti-dementia (Jeong *et al.* 2009; Zhao *et al.* 2008), and so on. With the interests in the synthesis of allocryptopine derivatives with biological activity, we report here the synthesis and crystal structure of the title compound, (I).

As shown in Fig. 1, the molecule of the title compound is characterized by the presence of a ten-membered ring (hexahydrodibenzo*[c,g]*azecine) with a methylated tertiary nitrogen atom and a hydroxyl group fused to two aryl moieties. In general, the title compound have two oxygenated substituents on the benzene ring and two methoxyl on the other benzene ring. Benzene rings C1/C2/C3/C4/C5/C6 and C10/C11/C16/C17/C18/C19 are inclined with respect to one another with a dihedral angle of 23.16 (5)°.

In the crystal structure, two adjacent molecules are linked by weak intermolecular O—H···O or C—H···O hydrogen bond into a one-dimension chain along *b* axis. These chains are further connected by C—H···π interaction into two-dimension sheets.

### Experimental

The title compound was synthesized according to the literature procedure (Wada *et al.* 2007), and colourless blocks of (I) were obtained from a solution in methanol by slow evaporation at room temperature.

### Refinement

H atoms were positioned geometrically and treated as riding, with C—H bond lengths constrained to 0.93 (aromatic CH), or 0.97 Å (methylene CH_2_), or 0.96Å (methyl CH_3_), and O—H = 0.82 Å, and with U_iso_(H) = 1.2U_eq_(C) or U_iso_(H) = 1.5Uc_eq_(O).

### Crystal data

**Table:**

C_21_H_25_NO_5_	*F*(000) = 792
*M**_r_* = 371.42	*D*_x_ = 1.261 Mg m^−^^3^
Monoclinic, *P*2_1_/*c*	Mo *K*α radiation, λ = 0.71073 Å
*a* = 14.2557 (19) Å	Cell parameters from 3665 reflections
*b* = 9.3705 (13) Å	θ = 2.6–22.6°
*c* = 15.278 (2) Å	µ = 0.09 mm^−^^1^
β = 106.601 (2)°	*T* = 296 K
*V* = 1955.8 (5) Å^3^	Block, colourless
*Z* = 4	0.45 × 0.24 × 0.21 mm

### Data collection

**Table:**

Bruker SMART APEX II CCD diffractometer	3646 independent reflections
Radiation source: fine-focus sealed tube	2766 reflections with *I* > 2σ(*I*)
graphite	*R*_int_ = 0.026
phi and ω scans	θ_max_ = 25.5°, θ_min_ = 2.6°
Absorption correction: multi-scan (*SADABS*; Sheldrick, 1996)	*h* = −17→17
*T*_min_ = 0.961, *T*_max_ = 0.981	*k* = −11→11
14189 measured reflections	*l* = −18→18

### Refinement

**Table:**

Refinement on *F*^2^	Primary atom site location: structure-invariant direct methods
Least-squares matrix: full	Secondary atom site location: difference Fourier map
*R*[*F*^2^ > 2σ(*F*^2^)] = 0.041	Hydrogen site location: inferred from neighbouring sites
*wR*(*F*^2^) = 0.110	H-atom parameters constrained
*S* = 1.02	*w* = 1/[σ^2^(*F*_o_^2^) + (0.0526*P*)^2^ + 0.4075*P*] where *P* = (*F*_o_^2^ + 2*F*_c_^2^)/3
3646 reflections	(Δ/σ)_max_ = 0.001
248 parameters	Δρ_max_ = 0.18 e Å^−^^3^
0 restraints	Δρ_min_ = −0.21 e Å^−^^3^

### Special details

**Table:**

Geometry. All e.s.d.'s (except the e.s.d. in the dihedral angle between two l.s. planes)are estimated using the full covariance matrix. The cell e.s.d.'s are takeninto account individually in the estimation of e.s.d.'s in distances, anglesand torsion angles; correlations between e.s.d.'s in cell parameters are onlyused when they are defined by crystal symmetry. An approximate (isotropic)treatment of cell e.s.d.'s is used for estimating e.s.d.'s involving l.s. planes.
Refinement. Refinement of *F*^2^ against ALL reflections. The weighted *R*-factor *wR* andgoodness of fit *S* are based on *F*^2^, conventional *R*-factors *R* are basedon *F*, with *F* set to zero for negative *F*^2^. The threshold expression of*F*^2^ > σ(*F*^2^) is used only for calculating *R*-factors(gt) *etc*. and isnot relevant to the choice of reflections for refinement. *R*-factors basedon *F*^2^ are statistically about twice as large as those based on *F*, and *R*-factors based on ALL data will be even larger.

### Fractional atomic coordinates and isotropic or equivalent isotropic displacement parameters (Å^2^)

**Table:**

	*x*	*y*	*z*	*U*_iso_*/*U*_eq_	
C1	0.27622 (11)	0.13752 (17)	0.24521 (10)	0.0402 (4)	
C2	0.32459 (11)	0.17612 (18)	0.18061 (11)	0.0460 (4)	
H2	0.2954	0.1612	0.1186	0.055*	
C3	0.41532 (12)	0.2359 (2)	0.21064 (13)	0.0528 (4)	
C4	0.46009 (13)	0.2568 (2)	0.30202 (14)	0.0592 (5)	
C5	0.41615 (12)	0.2188 (2)	0.36684 (13)	0.0588 (5)	
H5	0.4478	0.2329	0.4285	0.071*	
C6	0.32187 (11)	0.15767 (19)	0.33900 (11)	0.0464 (4)	
C7	0.55723 (15)	0.3447 (3)	0.22467 (16)	0.0776 (6)	
H7A	0.6186	0.3106	0.2168	0.093*	
H7B	0.5539	0.4471	0.2151	0.093*	
C8	0.17251 (11)	0.08232 (16)	0.20842 (9)	0.0375 (3)	
H8	0.1545	0.0280	0.2560	0.045*	
C9	0.10207 (11)	0.20855 (17)	0.17812 (11)	0.0410 (4)	
H9A	0.1234	0.2850	0.2221	0.049*	
H9B	0.1079	0.2429	0.1200	0.049*	
C10	−0.00464 (11)	0.17974 (16)	0.16778 (10)	0.0381 (3)	
C11	−0.04578 (11)	0.20517 (16)	0.23982 (10)	0.0381 (3)	
C12	0.01951 (11)	0.25595 (18)	0.33146 (11)	0.0444 (4)	
H12A	0.0477	0.3479	0.3243	0.053*	
H12B	−0.0189	0.2670	0.3742	0.053*	
C13	0.19042 (11)	0.2073 (2)	0.42234 (11)	0.0507 (4)	
H13A	0.1902	0.2099	0.4857	0.061*	
H13B	0.1988	0.3042	0.4035	0.061*	
C14	0.27663 (12)	0.1155 (2)	0.41377 (11)	0.0530 (5)	
H14A	0.3274	0.1183	0.4717	0.064*	
H14B	0.2544	0.0174	0.4034	0.064*	
C15	0.06335 (14)	0.0326 (2)	0.40923 (13)	0.0650 (5)	
H15A	0.1137	−0.0388	0.4255	0.098*	
H15B	0.0059	−0.0073	0.3672	0.098*	
H15C	0.0478	0.0646	0.4631	0.098*	
C16	−0.14540 (11)	0.18373 (17)	0.22552 (10)	0.0410 (4)	
C17	−0.20575 (11)	0.13956 (18)	0.14017 (11)	0.0452 (4)	
C18	−0.16472 (12)	0.11260 (18)	0.07058 (11)	0.0463 (4)	
H18	−0.2036	0.0809	0.0142	0.056*	
C19	−0.06541 (12)	0.13279 (17)	0.08482 (10)	0.0430 (4)	
H19	−0.0385	0.1143	0.0372	0.052*	
C20	−0.22408 (15)	0.0958 (2)	0.33403 (14)	0.0674 (5)	
H20A	−0.2690	0.0419	0.2868	0.101*	
H20B	−0.2569	0.1293	0.3769	0.101*	
H20C	−0.1700	0.0361	0.3650	0.101*	
C21	−0.36808 (14)	0.0889 (3)	0.04815 (15)	0.0854 (7)	
H21A	−0.3646	0.1587	0.0032	0.128*	
H21B	−0.4337	0.0846	0.0529	0.128*	
H21C	−0.3498	−0.0028	0.0303	0.128*	
N1	0.09746 (9)	0.15187 (14)	0.36674 (8)	0.0403 (3)	
O1	0.55278 (10)	0.3128 (2)	0.31403 (11)	0.0870 (5)	
O2	0.47711 (10)	0.27719 (17)	0.16005 (10)	0.0764 (4)	
O3	0.16204 (8)	−0.00602 (12)	0.12969 (7)	0.0458 (3)	
H3	0.1735	−0.0892	0.1458	0.069*	
O4	−0.30345 (8)	0.12764 (16)	0.13363 (8)	0.0642 (4)	
O5	−0.18857 (8)	0.21548 (12)	0.29420 (7)	0.0497 (3)	

### Atomic displacement parameters (Å^2^)

**Table:**

	*U*^11^	*U*^22^	*U*^33^	*U*^12^	*U*^13^	*U*^23^
C1	0.0378 (8)	0.0418 (9)	0.0418 (9)	0.0010 (7)	0.0127 (7)	−0.0015 (7)
C2	0.0419 (9)	0.0530 (10)	0.0431 (9)	0.0002 (7)	0.0121 (7)	0.0017 (7)
C3	0.0446 (10)	0.0587 (11)	0.0594 (11)	−0.0037 (8)	0.0216 (8)	0.0003 (9)
C4	0.0407 (9)	0.0668 (12)	0.0694 (12)	−0.0113 (9)	0.0147 (9)	−0.0111 (10)
C5	0.0432 (10)	0.0793 (14)	0.0511 (10)	−0.0077 (9)	0.0089 (8)	−0.0145 (9)
C6	0.0395 (9)	0.0553 (10)	0.0428 (9)	0.0013 (7)	0.0091 (7)	−0.0051 (8)
C7	0.0530 (12)	0.0853 (16)	0.0995 (17)	−0.0185 (11)	0.0298 (12)	−0.0081 (13)
C8	0.0417 (8)	0.0422 (9)	0.0301 (7)	−0.0026 (7)	0.0125 (6)	−0.0025 (6)
C9	0.0429 (9)	0.0425 (9)	0.0389 (8)	−0.0011 (7)	0.0140 (7)	0.0029 (7)
C10	0.0408 (8)	0.0357 (8)	0.0375 (8)	0.0027 (6)	0.0111 (6)	0.0027 (6)
C11	0.0407 (8)	0.0346 (8)	0.0381 (8)	0.0007 (6)	0.0095 (7)	−0.0015 (6)
C12	0.0430 (9)	0.0473 (9)	0.0436 (9)	−0.0029 (7)	0.0136 (7)	−0.0117 (7)
C13	0.0456 (9)	0.0690 (12)	0.0369 (8)	−0.0084 (8)	0.0108 (7)	−0.0137 (8)
C14	0.0440 (9)	0.0768 (13)	0.0347 (8)	−0.0011 (9)	0.0057 (7)	−0.0011 (8)
C15	0.0613 (11)	0.0714 (13)	0.0612 (12)	−0.0116 (10)	0.0156 (9)	0.0148 (10)
C16	0.0408 (8)	0.0424 (9)	0.0413 (9)	0.0025 (7)	0.0142 (7)	−0.0021 (7)
C17	0.0376 (9)	0.0493 (10)	0.0456 (9)	0.0016 (7)	0.0069 (7)	−0.0019 (7)
C18	0.0458 (9)	0.0531 (10)	0.0350 (8)	0.0012 (8)	0.0036 (7)	−0.0040 (7)
C19	0.0478 (9)	0.0462 (9)	0.0357 (8)	0.0048 (7)	0.0130 (7)	0.0005 (7)
C20	0.0694 (13)	0.0749 (14)	0.0672 (12)	−0.0005 (11)	0.0347 (10)	0.0095 (10)
C21	0.0436 (11)	0.128 (2)	0.0719 (14)	−0.0027 (12)	−0.0039 (10)	−0.0165 (14)
N1	0.0398 (7)	0.0478 (8)	0.0326 (6)	−0.0057 (6)	0.0095 (5)	−0.0040 (6)
O1	0.0522 (8)	0.1244 (14)	0.0852 (11)	−0.0369 (9)	0.0208 (7)	−0.0178 (10)
O2	0.0563 (8)	0.1040 (12)	0.0760 (9)	−0.0229 (8)	0.0303 (7)	0.0018 (8)
O3	0.0541 (7)	0.0482 (7)	0.0363 (6)	−0.0031 (6)	0.0150 (5)	−0.0074 (5)
O4	0.0363 (6)	0.0958 (10)	0.0561 (7)	−0.0040 (6)	0.0062 (5)	−0.0098 (7)
O5	0.0496 (7)	0.0551 (7)	0.0499 (7)	−0.0007 (5)	0.0229 (5)	−0.0060 (5)

### Geometric parameters (Å, °)

**Table:**

C1—C2	1.403 (2)	C12—H12B	0.9700
C1—C6	1.407 (2)	C13—N1	1.4506 (19)
C1—C8	1.515 (2)	C13—C14	1.536 (2)
C2—C3	1.363 (2)	C13—H13A	0.9700
C2—H2	0.9300	C13—H13B	0.9700
C3—C4	1.373 (3)	C14—H14A	0.9700
C3—O2	1.383 (2)	C14—H14B	0.9700
C4—C5	1.362 (3)	C15—N1	1.445 (2)
C4—O1	1.384 (2)	C15—H15A	0.9600
C5—C6	1.410 (2)	C15—H15B	0.9600
C5—H5	0.9300	C15—H15C	0.9600
C6—C14	1.516 (2)	C16—O5	1.3920 (18)
C7—O1	1.417 (3)	C16—C17	1.404 (2)
C7—O2	1.427 (2)	C17—O4	1.3720 (19)
C7—H7A	0.9700	C17—C18	1.375 (2)
C7—H7B	0.9700	C18—C19	1.383 (2)
C8—O3	1.4323 (17)	C18—H18	0.9300
C8—C9	1.535 (2)	C19—H19	0.9300
C8—H8	0.9800	C20—O5	1.435 (2)
C9—C10	1.508 (2)	C20—H20A	0.9600
C9—H9A	0.9700	C20—H20B	0.9600
C9—H9B	0.9700	C20—H20C	0.9600
C10—C19	1.387 (2)	C21—O4	1.414 (2)
C10—C11	1.407 (2)	C21—H21A	0.9600
C11—C16	1.388 (2)	C21—H21B	0.9600
C11—C12	1.519 (2)	C21—H21C	0.9600
C12—N1	1.461 (2)	O3—H3	0.8200
C12—H12A	0.9700		
			
C2—C1—C6	120.30 (14)	N1—C13—H13A	109.3
C2—C1—C8	116.79 (13)	C14—C13—H13A	109.3
C6—C1—C8	122.82 (13)	N1—C13—H13B	109.3
C3—C2—C1	118.61 (15)	C14—C13—H13B	109.3
C3—C2—H2	120.7	H13A—C13—H13B	108.0
C1—C2—H2	120.7	C6—C14—C13	116.17 (15)
C2—C3—C4	121.44 (16)	C6—C14—H14A	108.2
C2—C3—O2	128.43 (17)	C13—C14—H14A	108.2
C4—C3—O2	110.06 (15)	C6—C14—H14B	108.2
C5—C4—C3	121.68 (16)	C13—C14—H14B	108.2
C5—C4—O1	128.52 (18)	H14A—C14—H14B	107.4
C3—C4—O1	109.74 (17)	N1—C15—H15A	109.5
C4—C5—C6	118.93 (16)	N1—C15—H15B	109.5
C4—C5—H5	120.5	H15A—C15—H15B	109.5
C6—C5—H5	120.5	N1—C15—H15C	109.5
C1—C6—C5	119.03 (15)	H15A—C15—H15C	109.5
C1—C6—C14	124.04 (14)	H15B—C15—H15C	109.5
C5—C6—C14	116.92 (14)	C11—C16—O5	120.21 (13)
O1—C7—O2	109.03 (15)	C11—C16—C17	121.11 (14)
O1—C7—H7A	109.9	O5—C16—C17	118.53 (13)
O2—C7—H7A	109.9	O4—C17—C18	125.15 (14)
O1—C7—H7B	109.9	O4—C17—C16	115.57 (14)
O2—C7—H7B	109.9	C18—C17—C16	119.28 (14)
H7A—C7—H7B	108.3	C17—C18—C19	119.72 (14)
O3—C8—C1	111.43 (12)	C17—C18—H18	120.1
O3—C8—C9	106.85 (12)	C19—C18—H18	120.1
C1—C8—C9	109.50 (13)	C18—C19—C10	122.10 (14)
O3—C8—H8	109.7	C18—C19—H19	118.9
C1—C8—H8	109.7	C10—C19—H19	118.9
C9—C8—H8	109.7	O5—C20—H20A	109.5
C10—C9—C8	116.55 (13)	O5—C20—H20B	109.5
C10—C9—H9A	108.2	H20A—C20—H20B	109.5
C8—C9—H9A	108.2	O5—C20—H20C	109.5
C10—C9—H9B	108.2	H20A—C20—H20C	109.5
C8—C9—H9B	108.2	H20B—C20—H20C	109.5
H9A—C9—H9B	107.3	O4—C21—H21A	109.5
C19—C10—C11	118.51 (14)	O4—C21—H21B	109.5
C19—C10—C9	120.04 (13)	H21A—C21—H21B	109.5
C11—C10—C9	121.40 (13)	O4—C21—H21C	109.5
C16—C11—C10	119.24 (13)	H21A—C21—H21C	109.5
C16—C11—C12	121.34 (13)	H21B—C21—H21C	109.5
C10—C11—C12	119.42 (13)	C15—N1—C13	112.48 (14)
N1—C12—C11	109.40 (12)	C15—N1—C12	111.35 (14)
N1—C12—H12A	109.8	C13—N1—C12	116.64 (13)
C11—C12—H12A	109.8	C4—O1—C7	104.83 (15)
N1—C12—H12B	109.8	C3—O2—C7	104.43 (15)
C11—C12—H12B	109.8	C8—O3—H3	109.5
H12A—C12—H12B	108.2	C17—O4—C21	117.92 (14)
N1—C13—C14	111.64 (14)	C16—O5—C20	115.97 (13)
			
C6—C1—C2—C3	−1.3 (2)	C1—C6—C14—C13	−71.7 (2)
C8—C1—C2—C3	175.33 (15)	C5—C6—C14—C13	109.25 (18)
C1—C2—C3—C4	0.9 (3)	N1—C13—C14—C6	90.26 (18)
C1—C2—C3—O2	177.59 (17)	C10—C11—C16—O5	−176.56 (13)
C2—C3—C4—C5	0.1 (3)	C12—C11—C16—O5	3.2 (2)
O2—C3—C4—C5	−177.16 (18)	C10—C11—C16—C17	−1.1 (2)
C2—C3—C4—O1	177.33 (17)	C12—C11—C16—C17	178.70 (15)
O2—C3—C4—O1	0.1 (2)	C11—C16—C17—O4	−177.48 (14)
C3—C4—C5—C6	−0.6 (3)	O5—C16—C17—O4	−1.9 (2)
O1—C4—C5—C6	−177.3 (2)	C11—C16—C17—C18	2.2 (2)
C2—C1—C6—C5	0.8 (3)	O5—C16—C17—C18	177.73 (14)
C8—C1—C6—C5	−175.64 (15)	O4—C17—C18—C19	177.98 (16)
C2—C1—C6—C14	−178.21 (16)	C16—C17—C18—C19	−1.7 (2)
C8—C1—C6—C14	5.3 (3)	C17—C18—C19—C10	0.1 (3)
C4—C5—C6—C1	0.2 (3)	C11—C10—C19—C18	1.0 (2)
C4—C5—C6—C14	179.25 (17)	C9—C10—C19—C18	−176.33 (15)
C2—C1—C8—O3	38.31 (19)	C14—C13—N1—C15	79.54 (18)
C6—C1—C8—O3	−145.11 (15)	C14—C13—N1—C12	−150.04 (14)
C2—C1—C8—C9	−79.68 (17)	C11—C12—N1—C15	−80.48 (17)
C6—C1—C8—C9	96.90 (17)	C11—C12—N1—C13	148.58 (13)
O3—C8—C9—C10	77.80 (16)	C5—C4—O1—C7	−174.7 (2)
C1—C8—C9—C10	−161.39 (13)	C3—C4—O1—C7	8.3 (2)
C8—C9—C10—C19	−89.37 (18)	O2—C7—O1—C4	−13.5 (2)
C8—C9—C10—C11	93.35 (17)	C2—C3—O2—C7	174.7 (2)
C19—C10—C11—C16	−0.5 (2)	C4—C3—O2—C7	−8.3 (2)
C9—C10—C11—C16	176.82 (14)	O1—C7—O2—C3	13.6 (2)
C19—C10—C11—C12	179.70 (14)	C18—C17—O4—C21	−2.1 (3)
C9—C10—C11—C12	−3.0 (2)	C16—C17—O4—C21	177.58 (18)
C16—C11—C12—N1	121.81 (15)	C11—C16—O5—C20	−112.30 (17)
C10—C11—C12—N1	−58.39 (19)	C17—C16—O5—C20	72.12 (19)

### Hydrogen-bond geometry (Å, °)

**Table:**

Cg2 is the centroid of the C1–C6 benzene ring.

**Table:**

*D*—H···*A*	*D*—H	H···*A*	*D*···*A*	*D*—H···*A*
O3—H3···O5^i^	0.82	2.03	2.8380 (16)	168
C7—H7A···O4^ii^	0.97	2.57	3.405 (3)	144
C18—H18···O3^iii^	0.93	2.53	3.229 (2)	132
C7—H7B···Cg2^iv^	0.97	2.57	3.464 (3)	153

Symmetry codes: (i) −*x*, *y*−1/2, −*z*+1/2; (ii) *x*+1, *y*, *z*; (iii) −*x*, −*y*, −*z*; (iv) −*x*+1, *y*+1/2, −*z*+1/2.
